# Energy Metabolism during Repeated Sets of Leg Press Exercise Leading to Failure or Not

**DOI:** 10.1371/journal.pone.0040621

**Published:** 2012-07-13

**Authors:** Esteban M. Gorostiaga, Ion Navarro-Amézqueta, José A. L. Calbet, Ylva Hellsten, Roser Cusso, Mario Guerrero, Cristina Granados, Miriam González-Izal, Javier Ibañez, Mikel Izquierdo

**Affiliations:** 1 Studies, Research and Sport Medicine Center, Government of Navarre, Pamplona, Spain; 2 Department of Physical Education, University of Las Palmas de Gran Canaria, Las Palmas de Gran Canaria, Spain; 3 Molecular Physiology Group, Section of Human Physiology, Department of Exercise and Sport Sciences, Copenhagen Muscle Research Center, University of Copenhagen, Copenhagen, Denmark; 4 Department of Physiological Sciences I, Institut d’Investigacions Biomèdiques August Pi i Sunyer (IDIBAPS), University of Barcelona, Barcelona, Spain; University of Zaragoza, Spain

## Abstract

This investigation examined the influence of the number of repetitions per set on power output and muscle metabolism during leg press exercise. Six trained men (age 34±6 yr) randomly performed either 5 sets of 10 repetitions (10REP), or 10 sets of 5 repetitions (5REP) of bilateral leg press exercise, with the same initial load and rest intervals between sets. Muscle biopsies (vastus lateralis) were taken before the first set, and after the first and the final sets. Compared with 5REP, 10REP resulted in a markedly greater decrease (P<0.05) of the power output, muscle PCr and ATP content, and markedly higher (P<0.05) levels of muscle lactate and IMP. Significant correlations (P<0.01) were observed between changes in muscle PCr and muscle lactate (R^2^ = 0.46), between changes in muscle PCr and IMP (R^2^ = 0.44) as well as between changes in power output and changes in muscle ATP (R^2^ = 0.59) and lactate (R^2^ = 0.64) levels. Reducing the number of repetitions per set by 50% causes a lower disruption to the energy balance in the muscle. The correlations suggest that the changes in PCr and muscle lactate mainly occur simultaneously during exercise, whereas IMP only accumulates when PCr levels are low. The decrease in ATP stores may contribute to fatigue.

## Introduction

Regular resistance exercise is an essential component of effective intervention programs designed to improve strength in athletes and adults with chronic diseases and disabilities [1], [2]. The response to resistance training program depends ultimately on pronounced metabolic and morphological adaptations of multiple cellular functions which depend to great extent on changes of a complex signaling network that is involved during each training session in response to contractile activity [3], [4].

Whilst changes in muscle metabolites and power output during exhausting and non exhausting heavy intermittent cycling [5], [6], running [7] or isometric knee extension [8] exercises are well characterized, little is known on substrate utilization and metabolic demand during consecutive sets of exhausting compared with non exhausting high-intensity dynamic resistance exercise. The adaptive response to strength training may be different when training leads to failure is compared to when it does not lead to failure, as different degrees of fatigue and muscle metabolite accumulation are elicited by the training [9]. The purpose of this study was, therefore, to investigate the influence of the number of repetitions per set (leading to failure or not) on changes in muscle and blood metabolites and power output during high-intensity bilateral leg press exercise performed with the same initial load (∼83% 1RM) in young trained men, while simultaneously examining the power output and fatigue developed throughout the exercise. These two exercise sessions are traditionally used for reaching specific training goals. Thus, the “leading to failure” exercise is characterized by a progressively decrease in load and power throughout the repeated sets and is primarily used for increasing muscular strength and hypertrophy [10], [11]. The “not leading to failure” exercise is characterized by the maintenance of load and average power throughout the sets and is used primarily for optimizing muscle power development [12]. To the author’s knowledge, no study has analyzed the changes in muscle and blood metabolites during high-intensity dynamic exercise characterized by the maintenance of power output throughout the sets. Analyzing different conditions related to changes in power output may provide some clues to the understanding of the mechanisms by which the process of muscle contraction try to maintain an adequate function during dynamic resistance exercise [13]. A second purpose of the study was to examine the relationship between the metabolic status of muscle and changes in power output. This kind of examination may enhance the understanding of factors that limit fatigue during leg press exercise, and thus give an indication of the regulation of the metabolic pathways and of how the anaerobic mechanisms are interrelated during dynamic resistance exercise in man.

## Materials and Methods

### Subjects

Six healthy male volunteers participated in the study. Their mean (±SD) age, height, body mass, body mass index, estimated maximal oxygen uptake (VO_2_max) in cycle ergometer and maximal strength (1RM) during bilateral leg press exercise were 34±6 years, 179±5 cm, 74.5±7.2 kg, 23.3±1.7 Kg·m^−2^, 57.1±4.9 ml·kg^−1^·min^−1^ and 199±43 kg, respectively. All were trained athletes, mainly in endurance events, but none trained for competition. The mean percentage of slow twitch (ST) fibers for these subjects was 65±12%. The subjects were thoroughly informed of the purpose, nature, practical details and possible risks associated with the experiment, as well as the right to terminate participation at will, before they gave their voluntary written informed consent to participate. A medical examination was also completed by a physician. The present study is part of a project that has examined the metabolic, neural and training effects of leg press exercise and has been approved by the Institutional Review Committee of the Instituto Navarro del Deporte, according to the Declaration of Helsinki.

### Experimental Protocol and Design

This study was designed to examine the influence of the number of repetitions per set (leading vs not leading to repetition failure) in changes in muscle metabolites and power output during high-intensity bilateral leg press exercise. To eliminate any possible effect of confounding factors, several variables such as initial load and total number of repetitions were controlled by equating their values between both exercise sessions. Each subject participated in two experiments on separate days, on which they performed 50 repetitions with the same initial load. This initial load (i.e., 154±31 Kg or 83% 1RM, the heaviest load that could be correctly pressed only once using the correct technique) was the greatest weight which it was possible to complete 10 repetitions to failure (10RM) of leg press exercise. Failure was indicated by the inability to complete the next repetition. On one experimental day (“leading to failure protocol”) the subjects performed 5 sets of 10 repetitions to failure (10REP), separated by 2 min of rest between each set. On this experimental day the subjects were able to finish all 10 repetitions at the initial load during the first set. Sometimes, however, the subjects could not lift the initial load during the following sets due to fatigue. Whenever subjects could not lift the load it was decreased by 15 Kg, thus allowing them to complete the experiment (50 repetitions). On the other experimental day (“not leading to failure protocol”), the subjects performed 10 sets of 5 repetitions not to failure (5REP) with the same initial load as that of (10REP), separated by 2 min of rest between each set. Five repetitions per set were chosen during this non failure experimental day because this is the maximal number of repetitions at which maximal power production can be maintained or slightly (10–15%) decreased during a set of 10 repetitions to failure in leg extension exercise [14]. In 5REP all subjects were able to finish the entire protocol with the initial load and to maintain the average power production throughout the repeated sets. In 10 REP, however, all subjects decreased the average power production throughout the sets.

### Intervention Period

All subjects participated in the two experiments in random order. Experiments were carried out at the same time of the day one to two months apart. No changes were observed in the subjects in maximal leg press strength (1RM) between the first (194±25 Kg) and the second (185±32 Kg) experimental day. To avoid disturbance of the subjects, they were instructed to record their normal diet for 48 hours prior the first experimental day and to repeat the same diet prior the second experimental day.

### Preliminary Tests

Several pre-test sessions took place during the 3 weeks preceding the experiments. First, the subjects were familiarized with the experimental testing procedures about 2 weeks beforehand. Second, two weeks before the first experiment the subjects participated in a control testing day where resistance-load verifications for 1RM were determined in the leg press exercise machine. Then, after at least 10 min rest, the subjects performed a maximal repetitive set until failure with the load that theoretically should produce 10 repetitions to fatigue (∼85% of 1RM). If the number of repetitions until failure was equal to 10, the load was defined as a 10RM and used during the experimental main tests. If the number of repetitions until failure was different from 10, several trials of a maximal repetitive set until failure were performed on different days with lower or higher loads during subsequent test sessions, in order to determine the load leading to failure in exactly 10 repetitions. Third, the maximum oxygen uptake (VO_2_max) of each subject was estimated [15] on a separate day by using a continuous incremental test until exhaustion on a friction-loaded cycle ergometer (Monark Ergomedic 818E, Varberg, Sweden). The first work load (60 W) was high enough to ensure that exhaustion would occur within 8–14 min, the load being increased by 30 W at the end of every min. Heart rate was continuously monitored throughout the test (15 s) with a cardiotachometer (Sportester Polar, Kempele, Finland). Average power output at exhaustion was 347±27 W.

### Main Tests

On the morning of the experiment, the subject arrived after a light breakfast and a 2 h fast period. On arrival at the laboratory subjects rested on a bed for 20 minutes so that small incisions could be made under local anesthesia (2 ml, 1% lidocaine) in the skin and fascia over the vastus lateralis muscle of one leg. The subjects then completed a warm-up period consisting of a set of 5 repetitions at 50%, three to four repetitions at 75% and 1 repetition at 90% of maximal bilateral leg press strength (1RM). Three to four subsequent attempts were made to determine the 1RM. The resting period between maximal attempts was always 2 min. After 10 min rest, a muscle biopsy (initial biopsy) was taken from the middle region of the muscle vastus lateralis (15 cm above the patella and approximately 3 cm below entry through the fascia) and after an arterialized blood sample was drawn from the earlobe, previously hyperemized with Finalgon® (Boehringer Ingelheim, Germany). Then they performed either 5 sets of 10 repetitions to failure (10REP) or 10 set of 5 repetitions not to failure (5REP) with the maximum load possible to achieve 10 repetitions during the first set (10RM). Subjects were instructed to always try to displace the weight as fast as possible. The duration of each repetition decomposed in its concentric and eccentric components was recorded. Repetitions were interspaced by ∼1-s pauses to prevent stretch-shortening cycle enhancement of performance. The power output of each repetition was monitored continuously and measured during the concentric phase of leg press action. Immediately (within 5–10 s) after the last repetition of the first set and immediately after the last repetition of the last set muscle biopsies were taken in all subjects. Additional arterialized blood samples were drawn after 16 and 45 min of recovery to determine the post-exercise uric acid blood concentration. All participants were highly motivated and strong verbal encouragement was given to all subjects to motivate them to perform each repetition maximally and as rapidly as possible. Subjects remained fasted throughout the tests.

### Equipment

The study was performed on a horizontal bilateral leg extension variable resistance machine (i.e. leg press action in a sitting position; Technogym, Gambettola, Italy). The sitting position was individually adjusted to minimize displacement between the lower back and backrest during muscular force exertion and, therefore, to avoid posture changes. Subjects were instructed to put their feet in the same position on the force platform. The exercise machine incorporated four force transducers on a foot platform located below the subject’s feet. The strain gauges recorded the applied force (N) to an accuracy of 1 Newton at 1000 Hz. The force platform and leg press plate all remained stationary throughout the lift, while the body moved away from the feet. In addition, a rotational encoder (Computer Optical Products Inc, California, USA) was attached to the weight plates to record the position and direction of the displacement to an accuracy of 0.2 mm at 1000 Hz. Customized software was used to calculate power (immediate product of displacement velocity and applied force) per repetition. After the end of the exercise, results were integrated over 1-ms intervals. The maximum 10-ms integral of applied force and displacement velocity during each repetition is referred to as “peak power output”. The average 10-ms integral of applied force and displacement velocity over the total concentric contraction time of each repetition is referred to as “mean power output”.

### Muscle Samples

Muscle biopsies were taken as described by Bergstrom [16] from the right leg on each occasion. Muscle samples were immediately frozen (in 5–10 s) in liquid nitrogen and stored at −80°C for subsequent metabolite assay and histochemical analysis, after being freed from visible fat and connective tissue. [16].

### Analysis

Muscle Phosphocreatine (PCr), creatine (Cr) and lactate, were analyzed by fluorometric analysis [17]. Skeletal muscle adenine nucleotides and inosine monophosphate (IMP) were analyzed by high-performance liquid chromatography (HPLC) [18]. All muscle metabolite concentrations are expressed as mmol·Kg^−1^ wet muscle. In addition, a piece of the biopsy taken before the first set was frozen separately, later used for serial cross-sectioning (10 µm) and stained for myofibrillar ATPase after alkaline and acid preincubation [19] for fiber classification into slow-twitch (ST) and fast-twitch (FT) fibers.

### Calculations

Cellular energy charge, a measure of the extent to which the total adenine nucleotide pool of the cell (ATP, ADP and AMP) is phosphorylated, was estimated using the following equation:
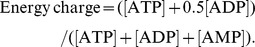



### Blood Samples

Capillary blood samples for determination of uric acid concentrations were obtained from a hyperemized earlobe at rest and 16 and 45 min after the end of the exercise protocol. According to the manufacturer’s instructions, after cleaning and puncturing a single 28.5–31.5-KL capillary sample was taken and placed over the strip (Reflotron uric acid) for an automatic reflectance photometry analysis (Reflotron; Boehringer Mannheim, Mannheim, Germany) within the first 2–3 min after obtaining the sample. The analyzer was calibrated (Reflotron Check) before every subject’s capillary samples analysis.

### Statistics

Standard statistical methods were used for the calculation of mean and standard deviation (SD). Student’s paired t-test was used for comparisons of analytic values during the two different experimental conditions in this study, whereas one-way analysis of variance for repeated measures was used to examine the differences in performance indexes and metabolite concentrations over time. When a significant F-value was achieved (P<0.05), the means were compared using a LSD post-hoc test. For the purposes of comparison the power output for the second 5 repetitions of each set was compared with that of the first 5 repetitions. Coefficient of determination (R^2^) was used to determine associations among variables taking both exercise conditions as a whole. Linear or non linear regressions were determined using either a linear or a second-degree polynomial form. A second-degree polynomial form was accepted if it resulted in a significant reduction in error variance as compared with the linear solution. Statistical significance was accepted at the P<0.05 level.

## Results

### Power and Force Production

During 5REP all the subjects were able to complete all the repetitions with the initially load assigned (154±31 Kg; 83±8% of 1RM). During 10REP, however, most of the participants were unable to complete all the repetitions with this starting load, due to failure. The load had to be reduced by 7.2±3.8% after 27±16 repetitions and was progressively reduced, reaching 85±12% (P<0.05) of the initial load at the last repetition. Average load during the 50 repetitions of 10REP was 6.1±6.3% lower (P<0.05) than during 5REP.

**Figure 1:**
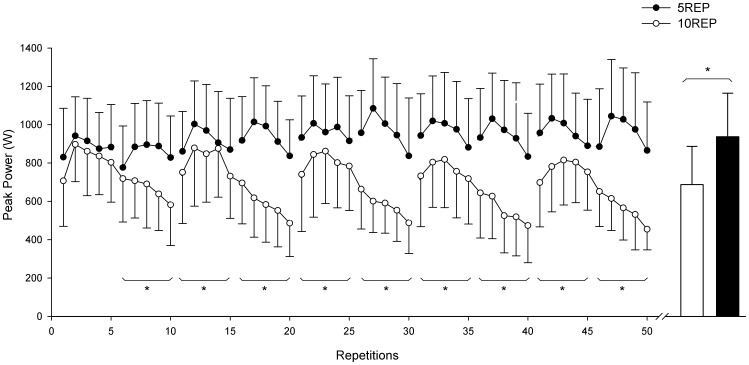
Peak power output profiles (average for n = 6 subjects) for each exercise during the two experimental conditions: when exercise was 5 sets of 10 repetitions to failure (10REP; open circles), and when exercise was 10 sets of 5 repetitions not to failure (5REP; filled circles). Boxes represent mean of the peak power output throughout 50 repetitions for 10REP and 5REP. *significant difference (P<0.05) between 10REP and 5REP (pooled from 5 to 5 repetitions). Values are means ± SD.

Average peak power during the first 5 repetitions of the first set was similar in both experimental conditions (Fig. 1). During each set of 5REP the highest value of average peak power was reached during the second or third repetition and thereafter average peak power decreased progressively (7–20%, P<0.05) between the second and the fifth repetition along each set. During each set of 10REP the highest value of average peak power was also reached during the second or third repetition and thereafter average peak power sharply decreased (35–45%, P<0.01) between the second and the tenth repetition along each set. During 10REP the magnitude of the decline in average peak power production between the first 5 repetitions of the first set and the last 5 repetitions of the last set was 33±19% (from 821±209 to 569±159 W; P<0.05). In contrast, during 5REP average peak power output in groups of 5 repetitions was maintained between sets. When both types of exercise were compared in groups of 5 repetitions, the average peak power from repetitions 6th to 50^th^ was 28±12% lower (P<0.01) during 10REP than during 5REP. In both experimental conditions, average mean power output changes paralleled those of peak power output.

### Muscle Metabolites

Table 1 shows muscle metabolite concentrations before the first set (initial biopsy) and immediately after the first and last set during both experimental days. Initial metabolite concentrations were within the normal range for human skeletal muscle. At the end of the 10REP exercise PCr stores were almost depleted (85% fall, P<0.05), whilst ATP (21%), energy charge (4%), and the total amount of adenine nucleotides pool (ATP + ADP + AMP) (20%) where reduced (P<0.05). Concomitantly, IMP and lactate where increased. In contrast to 10REP, 5REP resulted in markedly lower decrease of the muscle PCr (∼15% fall, P<0.05) and unchanged muscle ATP, IMP, energy charge and the total amount of adenine nucleotide pool concentrations, whereas muscle lactate was only slightly elevated above the initial levels. The changes observed in muscle ATP, total adenine nucleotide pool, IMP, PCr and lactate levels at the end of exercise were significantly higher (P<0.01–0.05) during 10REP compared with 5REP.

**Table 1:** Effects of leg press exercise on muscle adenine nucleotides, IMP, PCr, Cr, lactate and energy charge at before the first set, after the last repetition of the first set and after the last repetition of the last set, during 10REP and 5 REP exercise.

	10REP			5REP		
	Pre	Post 1^st^ set	Post last set	Pre	Post 1^st^ set	Post last set
**ATP**	6.46±0.56	6.42±0.57	4.90±0.39* †	6.58±0.35	6.19±0.59	6.09±0.41#
**ADP**	0.83±0.03	0.91±0.10	0.92±0.11	0.86±0.04	0.89±0.08	0.87±0.08
**AMP**	0.07±0.04	0.09±0.03	0.09±0.04	0.08±0.04	0.08±0.03#	0.08±0.03
**TAN**	7.37±0.59	7.42±0.67	5.91±0.44* †	7.52±0.36	7.16±0.66	7.04±0.49#
**IMP**	0.01±0.00	0.08±0.11	0.87±0.69*	0.01±0.00	0.01±0.00	0.01±0.02#
**PCr**	21.0±8.86	7.75±5.53	3.15±2.88*	19.5±4.06	11.68±7.82* ^#^	14.47±7.24* ^#^
**Cr**	8.93±4.96	25.45±3.80	22.90±6.89*	8.40±3.25*	16.97±6.33*	15.57±5.01*
**PCr + Cr**	29.91±5.19	34.55±6.23	26.06±8.44	27.90±3.65	30.56±6.19	30.14±8.46
**La**	1.70±1.18	17.20±3.50*	25.01±8.09*	2.02±1.05	7.10±2.54* ^#^	5.80±4.62#
**Energy Charge**	0.933±0.006	0.927±0.004*	0.909±0.014* †	0.932±0.007	0.927±0.006	0.928±0.006

Values are expressed as mean ± SD in mmol•kg^−1^ wet wt muscle, except energy charge; n = 4−6. TAN, total adenine nucleotides (ATP + ADP + AMP); IMP, Inosine 5′- monophosphate; PCr, Phosphocreatine; Cr, Creatine; La, Lactate. For calculations of energy charge see METHODS.

* significant difference (P<0.05) with pre exercise value.

† significant difference (P<0.05) with post first set value.

# significant difference (P<0.01–0.05) with 10REP exercise.

Compared with initial values, at the end of exercise the calculated ATP/ADP ratios decreased by 9% (P<0.05) in 5REP and by 30% (P<0.01) in 10REP (Table 2). The decrease of the ATP/ADP ratio in 10 REP was higher than the corresponding decrease observed in 5REP. The ATP/AMP ratio did not change at the end of 5REP but decreased (P<0.05) by 34% at the end of 10REP. Similarly, the ATP/IMP ratio did not change after 5REP but showed a pronounced decrease from 1049 to 13.4 (P<0.05) at the end of 10REP. No change occurred in the ADP/AMP ratio throughout exercises.

**Table 2:** Effects of leg press exercise on nucleotide metabolite ratios before the first set and during 10REP and 5REP exercise protocols.

	10REP			5REP		
	Pre	Post 1^st^ set	Post last set	Pre	Post 1^st^ set	Post last set
**ATP/ADP**	7.7±0.5	7.1±0.2	5.4±0.7* †	7.7±0.4	7.0±0.3*	7.0±0.4* #
**ATP/AMP**	100.5±29.3	77.8±19.7*	66.5±27.2*	91.2±30.6	88.4±24.8#	89.1±24.4#
**ADP/AMP**	12.9±3.6	10.9±2.7	12.1±3.9	11.8±3.7	12.6±3.3	12.7±3.3
**ATP/IMP**	1049.3±78.2	648.4±553.8	13.4±13.0*	1053.3±35.5	1055.3±23.2	773.2±406.2#

Values are expressed as mean ± SD.

* significant difference (P<0.05) with pre exercise value.

† significant difference (P<0.05) with post first set value.

# significant difference (P<0.01–0.05) with 10REP exercise.

### Blood Uric Acid

Peak blood uric acid concentration during recovery after the last set of 10REP (351±96 µmol·l^−1^) was 19% higher (P<0.05) than initial values (276±52 µmol·l^−1^). In 5REP the peak blood uric acid concentration after exercise (292±74 µmol·l^−1^) remained unchanged from initial values (286±701 µmol·l^−1^). The postexercise blood uric acid concentration value during 10REP was higher (P<0.05) than during 5REP.

### Relationships between Muscle Metabolites

**Figure 2:**
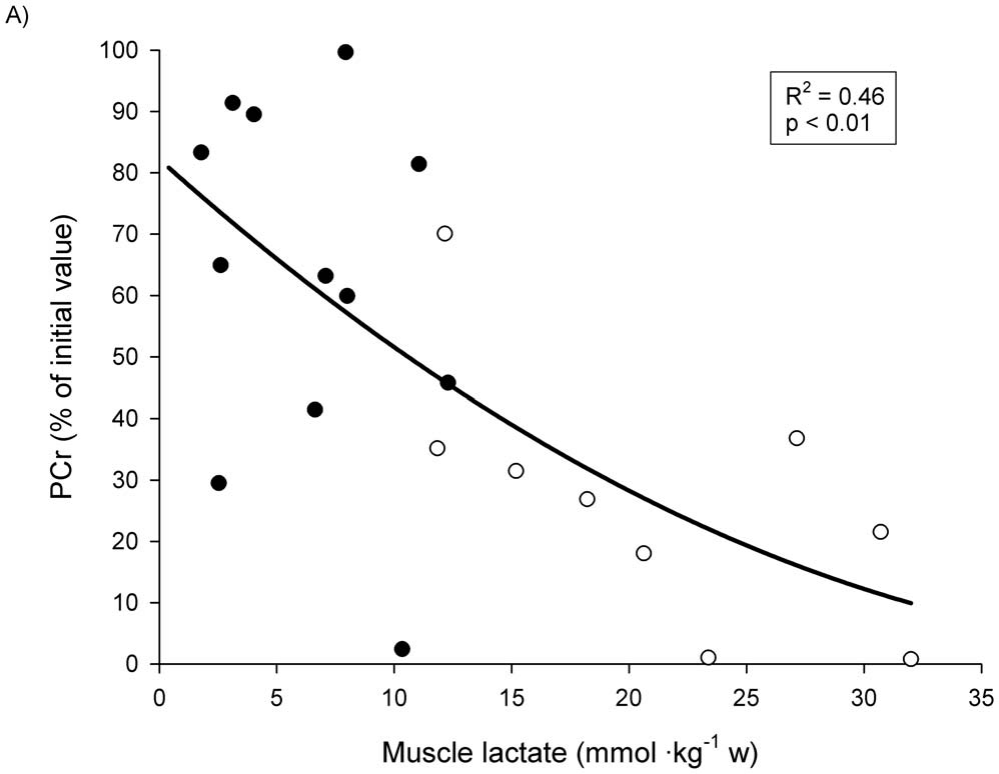
Individual relationship between muscle lactate concentrations and PCr concentrations (expressed in percent of initial value) (2A) and between PCr decreases (expressed in percent of initial value) and muscle IMP concentrations (2B), during 10REP (open circles), and 5REP (filled circles).

To examine the relationships between variables, both exercise conditions were taken as a whole. A significant curvilinear negative relationship was observed between muscle lactate content after the first and the last set and the corresponding levels of muscle PCr (in % of the initial levels) (R^2^ = 0.46, P<0.05) (Figure 2A). Similarly, a significant curvilinear relationship was observed between the relative decrease in muscle PCr levels (in % of the initial levels) and the levels of muscle IMP (R^2^ = 0.44, P<0.01) (Figure 2B). From these curvilinear relationships it can be estimated that a 60% decrease in PCr below rest values is required to elicit muscle IMP accumulation.

### Relationships between Muscle Metabolites and Changes in Power Output

**Figure 3:**
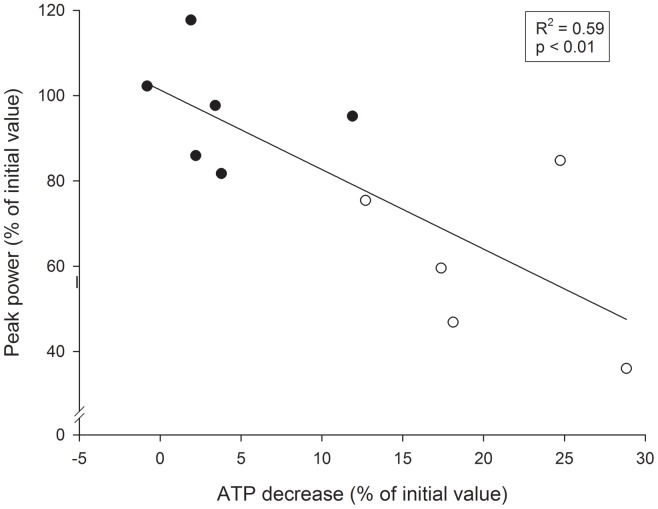
Individual relationships between the relative average peak power output changes (expressed in percent of initial value) between the first and the last two repetitions, and ATP decreases (expressed in percent of initial value), during 10REP exercise (open circles), and 5REP exercise (filled circles).

**Figure 4:**
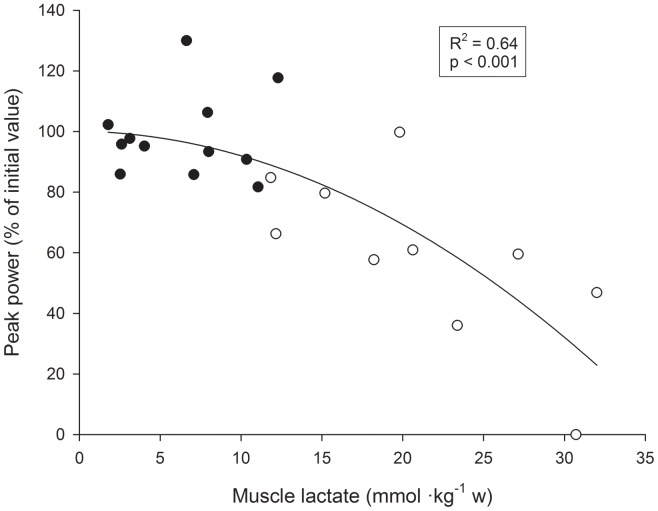
Individual relationships between the relative average peak power output changes (expressed in percent of initial value) between the first and the last two repetitions of the first set and between the first and last two repetitions of the exercise, and muscle lactate concentrations, during 10REP (open circles), and 5REP (filled circles).

A significant linear negative correlation was observed between the average changes in peak power output observed during the last two repetitions (expressed in percent of the initial two repetition values) and the decreases in ATP levels (expressed in percent of initial value) (R^2^ = 0.59, P<0.01) (Figure 3). Furthermore, a significant curvilinear negative correlation was observed between the average peak power output changes observed during the last two repetitions of the first and last sets (expressed in percent of the initial two repetition values) and the corresponding levels of muscle lactate (R^2^ = 0.64, P<0.01) (Figure 4). The curvilinear nature of the curve seems to indicate that when muscle lactate levels do not exceed the upper limit of 10–15 mmol·Kg^−1^ wet muscle, power output changes little from maximum values. However, when muscle lactate values exceed this upper level, the power output decreases sharply.

## Discussion

### Muscle Metabolism

The 10REP leg press exercise demanded a maximal effort from the subjects, as reflected by the marked decrease in power output that took place during the last 5 repetitions of each set. The use of this model of 10REP caused a state of energy deficiency and a decline in the phosphate potential resulting in a near-complete depletion of PCr stores, a significant reduction in ATP (21%) and the size of muscle total adenine nucleotide pool (TAN), and in marked increases in lactate and IMP accumulation in the muscle as well as high levels of uric acid in the blood. The elevated levels of muscle lactate indicate that anaerobic glycolysis is extensively activated during this type of exercise. The increased muscle IMP originates from degradation of ATP and appears to reflect the failure of ATP resynthesis to match ATP hydrolysis rates [13]. The elevated plasma accumulation of uric acid after 10RP suggests that IMP was not reaminated back to AMP after exercise, and that there instead was a dephosphorylation of IMP to inosine, and consequently hypoxanthine and uric acid [20]. The net result would have been a wasteful loss of purines from muscle [20] that would require replacement by either the purine nucleotide cycle or the novo synthesis [4]. The extent of anaerobic energy production and the fall in peak power output with 10REP is higher than the changes found previously in anabolic steroids user body–builders following an exercise regimen comprising 5 sets of 10RM each of front squats, back squats, leg presses and knee extensions [21]. However, it is qualitatively and quantitatively similar to what has been reported previously by others during and after high intensity intermittent cycling [5], [6], knee isometric [8], or isokinetic [22] exercises leading to exhaustion between 6 and 60s. These changes have been also associated with large reductions in muscle glycogen [6] and ATP [6], [22], particularly in Type II fibers [23], with the most pronounced changes being located in type IIx fibers [24], whereas blood and muscle pH can reach values as low as 7.1 [5] and 6.6 [25] respectively. However, the experimental protocol in previous studies was not the same as in the present study in which repetitive isoinertial contractions using leg press actions were used. Thus, it appears that 10REP causes a marked disruption to the energy balance in the muscle.

In contrast to 10 repetitions with a 10RM load (10REP), the energy balance was maintained during 5 repetitions with a 10RM load (5REP), despite the accumulation of sets. Thus, compared with 10REP, 5REP resulted in a markedly lower decrease in muscle PCr content (∼15% vs 80% fall) with only modest increases in muscle lactate and no measurable changes in muscle levels of ATP and IMP and in blood levels of uric acid. These minor metabolite changes observed in 5REP, despite a high ATP turnover rate, suggest that ATP synthesis matched ATP utilization and that cellular homeostasis was maintained, thereby demonstrating that the rate of AMP deamination was low [13]. Consequently, peak power output was maintained only during the 5REP exercise. The absence of blood uric acid increase during 5REP is in line with the finding of unchanged ATP levels. These results indicate that reducing the number of repetitions per set by 50% maintains power output and energy balance in the muscle throughout sets.

The changes in the ATP/ADP and ATP/AMP ratios during the contraction process have been widely studied because the relative levels of the adenine nucleotides are more important metabolic regulators for maintenance of adequate cellular functions than the absolute concentration of ATP [13]. At the end of 5REP the calculated ATP/ADP ratio was decreased by 9% compared with initial values, and was coincidental with no changes in the calculated energy charge, AMP, ATP/AMP and ATP/IMP ratios, with a 7–20% decrease in the average peak power between the second and the fifth repetition of each set, and with moderate decreases (30%) in PCr levels. The dichotomy between changes in the ATP/ADP ratio and changes in the ATP/AMP ratio during exercise is not without precedent. Thus, increases in the estimated muscle-free ADP content, without any changes in estimated free AMP, have been observed during 15 s sprint isokinetic cycling exercise [26] or after 10 minutes of cycling at 65% of maximal oxygen uptake [27], indicating that an slight initial increase in ADP availability does not displace the adenylaye kinase favouring the formation of AMP (ADP + ADP ↔ AMP + ATP), when the decrease in power production and the changes in muscle PCr or P_i_ levels are moderate. The activity of adenylate kinase was still low after 5REP, probably because the dephosphorylation of ADP to AMP is buffered by PCr [23]. In this situation the AMP-activated protein kinase (AMPK) may be only slightly activated by the increased ADP availability [28]. At the end of 10REP, however, a further decrease (30%) in the ATP/ADP ratio, higher to that seen after 5 REP, was accompanied by decreases in the ATP/AMP and ATP/IMP ratios, in parallel with further decreases (33%) in power production and with almost depleted PCr levels. The decreased ATP/AMP and ATP/IMP ratios indicate that, as opposed to 5REP, during 10REP the adenylate kinase and the AMP deaminase were significantly activated. The very low levels of PCr and the activation of adenylate kinase could amplify the activation of AMPK, that acts as a fuel-sensing enzyme monitoring cellular energy levels to prevent the catastrophic consequences of larger decreases in energy state [29]–[31]. This dual mechanism (initial decreases in ATP/ADP ratio followed by later decreases in ATP/AMP and ATP/IMP ratio) would allow AMPK to sense energy deficit progressively over a wide range of energy availability [28].

The described repetition-related differences in acute metabolic response to repeated sets of leg press exercise should reflect two different stimuli for training-induced adaptations occurring after heavy-resistance training. Thus, some studies have shown that high-intensity resistance training not to failure of the knee extensor muscles enables a favorable environment for achieving greater enhancements in maximal strength and power output compared with training to failure [12], [32], [33]. Taken together, the results of these studies and the present one suggest that a program of dynamic knee extension resistance exercise characterized by low metabolite accumulation and maintenance of cellular homeostasis and energy balance may be a more effective, efficient and safe option compared with training to failure designed to maximize fatigue/metabolite accumulation. Under this assumption, it would be time to replace the classical “no pain, no gain” training philosophy to a more rational and ecologically based “no pain, more gain” one.

### Relationships between Muscle Metabolites

Previous studies have shown that during fatiguing and not fatiguing isometric knee extension and cycling exercise average absolute PCr content changed curvilinearly and negatively with respect to the absolute muscle lactate content [34]. In the present study a significant curvilinear negative relationship was observed throughout the exercises between the percent decrease of muscle PCr and the corresponding muscle lactate content. This agrees with the findings of Karlsson and co-workers [35] during maximal and submaximal cycling exercise and with the close relationship between the logarithm of mass-action ratio of the creatine kinase reaction and muscle pH reported in men after isometric knee extension exercise [36]. Furthermore, it indicates that the changes between the PCr and lactate mainly occur simultaneously during exercise, supporting the observations of others that the anaerobic glycolysis is initiated in the muscle at the onset of heavy exercise [37]. In agreement with our results, accumulation of IMP matched quantitatively by a decline in intramuscular ATP has been reported during submaximal and maximal cycling exercise when PCr levels drop below 40% of the resting levels [13], [35], [38], [39].

### What Causes Fatigue during Consecutive Sets of Leg Press Exercise?

Several authors have suggested that the capacity to regenerate ATP at the required rates and thus decrease force and power production during short duration maximal exercise may by related to an inability to maintain the required rate of anaerobic ATP production from PCr and glycogen degradation, mainly in type II fibers [40], [41], a corresponding increase in inorganic phosphate (Pi) and its diprotonated form, H_2_PO^4−^, increases in [H^+^], alterations in Ca^2+^ transport [42], K^+^ efflux from the muscle [43] or impaired neuromuscular transmission or failure of membrane excitation [44]. In the present study the fall in power production during both exercises as a whole was strongly correlated to the fall in ATP stores and to the lactate levels in mixed muscle homogenates. The association between changes in power output and changes in muscle lactate accumulation are in agreement with the above mentioned studies. The association observed between the loss of ATP stores and the relative decline in power output supports the idea that ATP depletion in a small percentage of fibers may lead to their failure to power production [41]. This idea is in agreement with some studies reporting post-exercise ATP levels in individual fibers as low as 1 to 2.4 mmol·Kg^−1^ wet muscle following maximal knee extension [22], or isokinetic cycling [41] exercise. Hence, it is not unreasonable to suggest that some biochemical changes, such as a decrease in ATP stores and increases in lactate and by-products of ATP (H^+^, P_i_, ADP) of individual muscle fibers may contribute to fatigue during successive sets of leg press exercise.

### Study Limitations

This study had some limitations. First, it was characterized by a low number of experimental subjects. However, the strong statistically significant differences observed in muscle metabolites and power between the two exercises indicates that it is unlikely these differences occurred by chance. Future longitudinal biopsy studies should seek to recruit larger numbers of experimental subjects in order to reduce the potential risk of type II errors. Second, it may also be argued that the experimental setup could have been strengthened by matching the same load throughout sets and the total rest period between protocols (e.g. 1-min rest between sets in 5REP and 2-min rest between sets in 10REP). This would enable comparisons between two types of equivolumic exercise. It is obvious that, in this case, the magnitude of the differences between exercises would be lower than that observed in the present study. However, it has to be emphasized that the main goal was to compare metabolic and power changes during two types of resistance exercises traditionally used for reaching specific training outcomes (“hypertrophy” versus “optimal strength improvement”), but not to compare two equivolumic exercises. Finally, this study was performed in endurance trained men with a high proportion (65%) of type I fibers. Care should be taken when generalizing the results of this study to other populations (e.g. power athletes with high proportion of type II fibers). Despite these limitations, the findings do provide important and new information about the metabolic characteristics of two of the most popular high-intensity resistance exercises designed to increase muscular strength, power and hypertrophy in athletes and adults with chronic diseases and disabilities.

In summary, the main results of this study were, first, that reducing the number of repetitions during sets from ten to five while maintaining the same initial load and recovery periods in between induced markedly smaller demands on the high-energy phosphates system and on glycolytic energy supply, allowed ATP synthesis to match ATP utilization, to maintain power output and energy balance along the sets and to experience much less fatigue and discomfort during bilateral leg press exercise. Second, in mixed muscle homogenates the changes in muscle PCr during both exercises were correlated with muscle lactate and IMP content. These correlations suggest that the changes between the PCr and muscle lactate mainly occur simultaneously during exercise whereas IMP only accumulates when PCr levels are low. Finally, the fall in power production was strongly correlated to the fall in ATP stores and to the muscle lactate levels. This suggest that a decrease in ATP stores and increases in lactate and by-products of ATP (H^+^, P_i_, ADP) of individual muscle fibers may contribute to fatigue during successive sets of leg press exercise.
