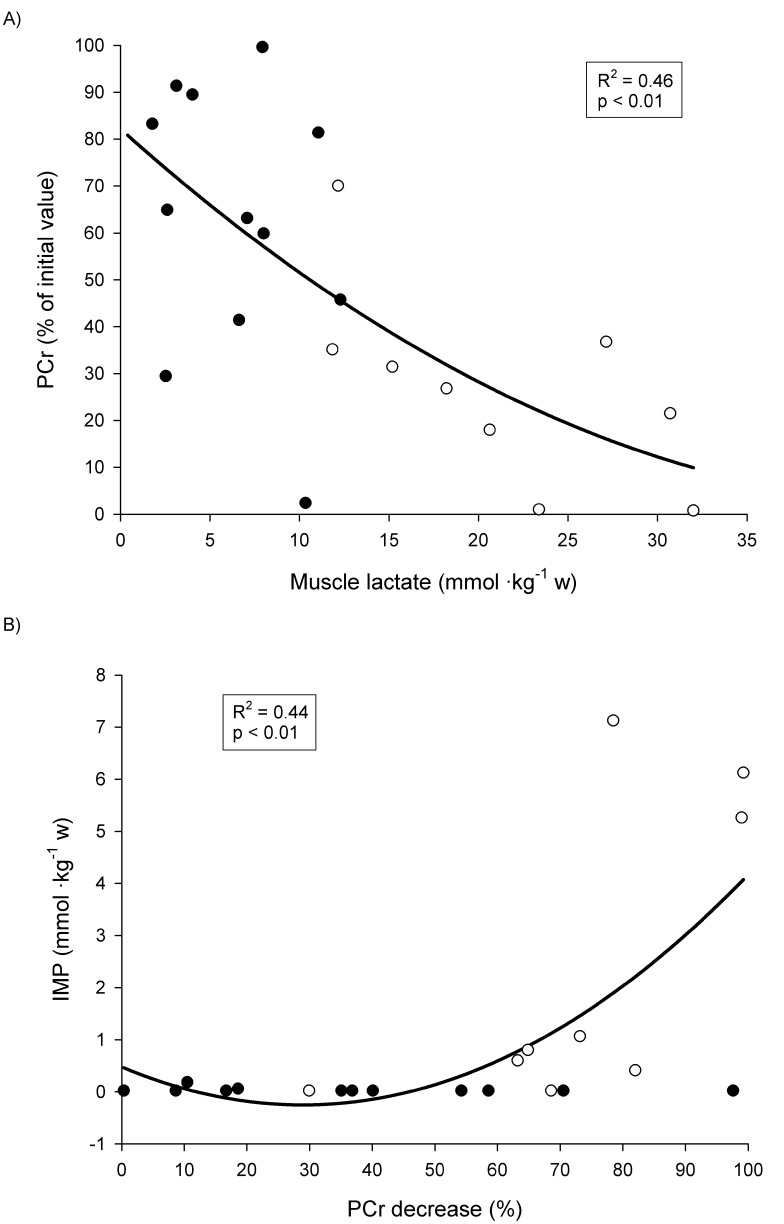# Correction: Energy Metabolism during Repeated Sets of Leg Press Exercise Leading to Failure or Not

**DOI:** 10.1371/annotation/ca21efee-84a1-4031-9dcd-224af64b7753

**Published:** 2013-01-17

**Authors:** Esteban M. Gorostiaga, Ion Navarro-Amézqueta, José A. L. Calbet, Ylva Hellsten, Roser Cusso, Mario Guerrero, Cristina Granados, Miriam González-Izal, Javier Ibañez, Mikel Izquierdo

In Figure 2, panel B is missing. Please see the correct Figure 2 here: 

**Figure:**